# Buffering the Breach: Examining the Three-Way Interaction Between Unit Climate Level, Strength, and Psychological Contract Breach

**DOI:** 10.3389/fpsyg.2019.00473

**Published:** 2019-03-05

**Authors:** Jos Akkermans, P. Matthijs Bal, Simon B. De Jong

**Affiliations:** ^1^School of Business and Economics, Vrije Universiteit Amsterdam, Amsterdam, Netherlands; ^2^Lincoln International Business School, University of Lincoln, Lincoln, United Kingdom; ^3^Department of Industrial Psychology and People Management, University of Johannesburg, Johannesburg, South Africa; ^4^School of Business and Economics, Maastricht University, Maastricht, Netherlands

**Keywords:** psychological contract breach, unit climate, work engagement, turnover intentions, social information processing (SIP) theory, conservation of resource theory (COR)

## Abstract

Despite the wealth of research showing that psychological contract breach (PCB) has negative outcomes for individuals, knowledge about the influence of the social context in which breaches are experienced is still scarce. This is surprising, as scholars have argued that work climates, such as when unit members are generally highly committed, could buffer an individual’s negative experiences at work. Yet, to date, the unit climate and PCB literatures have largely remained separated and our main goal is to integrate these fields. This is especially timely and relevant, because recent work in the unit climate literature indicates that merely looking at the average climate *level* might not be enough, because the climate’s *strength* (i.e., the agreement or homogeneity within the unit) could also provide important social cues. Building on these recent advances, we develop and test a theoretical framework which links both climate concepts to PCB. More specifically, we hypothesized that especially when all unit members are highly *and* homogeneously committed, an employee would reframe their PCB in such a way that it would less adversely affect work engagement and turnover intentions. Using data from 1,272 employees across 36 healthcare units, multilevel structural path analyses supported this three-way interaction. By answering recent calls for more “social PCB research” and integrating the unit climate and PCB literatures, we aim to provide guidance to scholars and practitioners who want to understand in more depth the social context’s influence on PCB.

## Introduction

The psychological contract has become a dominant framework for understanding how individuals care and “feel” about their organization and has been shown to affect important outcomes such as employee work engagement and turnover intentions (Halbesleben, 2010). The psychological contract refers to an employee’s perception about mutual obligations that exist between them and the organization, and when an employee perceives that the organization does not meet these obligations, psychological contract breach (PCB) occurs (Rousseau, 1989; Robinson and Rousseau, 1994). Yet, not every breach affects employees to the same extent (Morrison and Robinson, 1997) because various factors determine employee reactions to breach. Until recently, researchers predominantly focused on individual-level variables moderating PCB effects (e.g., Zhao et al., 2007; Bal et al., 2008), yet recently the social context has received more attention (e.g., Ho, 2005; Ho et al., 2006; Dabos and Rousseau, 2013; O’Leary-Kelly et al., 2014). This “social PCB research” can, in short, be divided into two main approaches. First, most of the social PCB research has analyzed PCB and its effects still at the individual-level, yet then assessed how individual perceptions of the social context moderated these effects (e.g., Restubog et al., 2015). Second, some emerging research has aggregated the psychological contract to higher levels, for example via introducing new “shared” concepts (e.g., Laulié and Tekleab, 2016).

Yet, these two streams of research have not yet been integrated theoretically and empirically, as thus far research has either looked at the team or only at individual employee perceptions of the social context. Hence, in the current study we will argue that it is important to take a multilevel perspective, and investigate how the social context (e.g., the commitment within a unit) affects individual employees in their responses to PCB. Moreover, existing research tends to focus on *average* levels of the social context (Restubog et al., 2015), whereby the underlying, often implicit, assumption is that only the shared/average level is deemed to matter. Differences and variations in perceptions in the unit are typically viewed as measurement error that needs to be filtered out. In the current study, we will theorize – and test empirically – the relevance of both average levels and variations in social context in relation to the effects of PCB. To do so, we will build a theoretical model on the basis of individual-level relationships of PCB with turnover intentions via work engagement (Halbesleben, 2010; Bailey et al., 2017). These attitudes represent key outcomes of PCB, since having an engaged workforce that is intent on remaining with the organization is very important in today’s dynamic, demanding, and increasingly globalized business world (Turnley et al., 2003; Cappelli and Keller, 2013).

Expanding upon that foundation, we will introduce our main contribution by integrating the unit climate literature (e.g., González-Romá et al., 2009) into the PCB literature. In short, we will argue that it is not only important to consider the mean *level* of a shared climate as perceived by members within a working unit, but also the *strength* of these perceptions, that is, the extent to which these climate perceptions vary among unit members. Moreover, we argue that especially *the combination* of climate level and climate strength is important (Cole et al., 2011). To do this, we argue that the affective commitment climate within the unit (hereafter shortened to unit commitment climate) is particularly relevant, given its centrality in prior PCB research (e.g., Guerrero et al., 2014; Tomprou et al., 2015; Hansen and Griep, 2016; Solinger et al., 2016). Commitment climate can be characterized as a dynamic summary of evaluative affect and a pledge to serve and enhance the organization’s purposes (cf. Solinger et al., 2015) and thus also fits our purposes as it captures how employees within a unit care and “feel” about their organization.

**FIGURE 1:**
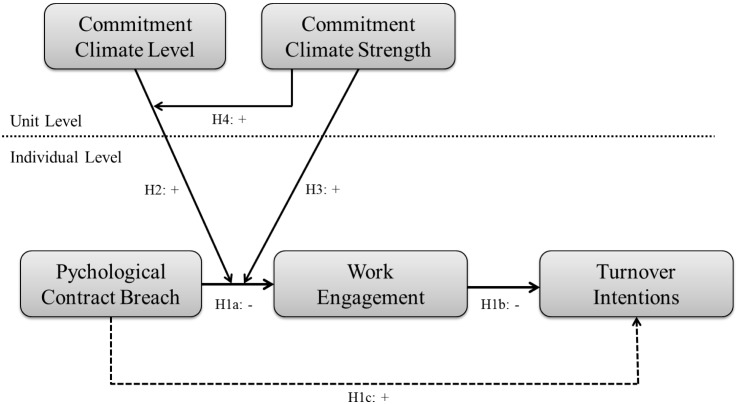
Research model.

Our conceptual model is depicted in Figure 1. First, we expect that PCB is associated with lower work engagement and then subsequently with higher turnover intentions of individual employees, thereby answering to calls for more replication of results in the (organizational) psychological and management literatures (e.g., Makel et al., 2012). Second, we expand on contemporary knowledge by investigating new cross-level relationships between unit-level climate and individual-level PCB. Specifically, by drawing on the unit climate literature, we expect two two-way interactions in which the unit’s affective commitment climate *level* and affective commitment climate *strength* each moderate the negative relation between employee PCB and work engagement. Finally, we expect a three-way interaction, in which the detrimental effects of PCB will especially be buffered when *both* affective commitment climate level and strength are high. Below we will first discuss our basic model, and then discuss the role of the unit context.

## Theory

### Psychological Contract Breach, Work Engagement, and Turnover Intentions

In this paper, the theoretical rationale underlying the relationships between PCB and work engagement – which is defined as a positive work-related state of fulfillment that at its core is characterized by vigor and dedication (Schaufeli et al., 2002; González-Romá et al., 2006) – is based on Conservation of Resources (COR) theory (Hobfoll, 2002). COR theory states that individuals are motivated to protect and replenish their resources, and that gaining resources can subsequently lead to gaining even more resources. Using this reasoning, Garcia et al. (2018) recently framed a PCB as a perceived or actual loss of resources. PCB can be characterized as the employee’s perception and cognitive evaluation regarding the extent to which the organization has failed to fulfill its promises or obligations (Robinson and Rousseau, 1994; Morrison and Robinson, 1997). As such, it reflects the employee’s perception of their organization not providing promised resources, and can therefore be considered a resource loss (Halbesleben, 2006; Bordia et al., 2014), which can potentially lead to a resource depletion process in which the initial loss of resources sets into motion a process of further resource losses. Accordingly, and in line with COR Theory, because work engagement has been shown to be predicted by the availability of job and personal resources (Bakker and Demerouti, 2017), and because PCB triggers a feeling of loss of resources, it is likely that PCB will be negatively associated with work engagement (cf. Rayton and Yalabik, 2014). We therefore expect that:

**Hypothesis 1a**: *PCB will be negatively related to work engagement.*

Work engagement, in turn, has been related to turnover intentions (Mobley, 1977; Schaufeli and Bakker, 2004). Continuing to draw on COR theory, we expect that highly engaged employees typically have gathered many resources in their work, and they show high levels of dedication in their jobs (Halbesleben and Wheeler, 2008). Starting a new job would imply that an engaged employee would have to “start over” and gather new resources, which is a high-risk situation. Because COR theory states that individuals tend to protect their available resources and avoid risks of losing them (Hobfoll, 2001), it is unlikely that engaged workers would want to leave their job as a result of the accumulated resources in their current job. Conversely, when PCB occurs, these accumulated resources are threatened and, as a result, the employee will be less strongly bound to their organization as they might just as easily build new resources elsewhere. In line with this reasoning, Podsakoff et al. (2007) showed in their meta-analysis that job-related attitudes, such as work engagement, can diminish turnover. Moreover, Halbesleben (2010) performed a meta-analysis on work engagement and showed that increased levels of work engagement can lead to lower turnover intentions. Taken together, we therefore expect that employees who are highly engaged will be less likely to want to leave their organization (Halbesleben and Wheeler, 2008). Hence, we formulate the following hypothesis:

**Hypothesis 1b**: *Work engagement will be negatively related to employee turnover intentions*.

Building on the above hypothesized relationships, we expect that work engagement is likely to mediate the relationship between PCB and turnover intentions. This is in line with COR theory (Halbesleben et al., 2014), as the initial experience of resource loss (i.e., PCB) will set into motion a resource depletion process that diminishes work engagement, which subsequently increases intentions to leave the organization. This argumentation is in line with prior research which theorized, and subsequently demonstrated, that PCB is related to behavioral intentions via their mediated relationships with job attitudes (e.g., Zhao et al., 2007). In addition, Parzefall and Hakanen (2010) argued and showed that a *high-quality* employment relationship (i.e., psychological contract fulfillment) leads to a positive affective–cognitive state of mind (i.e., work engagement), which then subsequently affects outcomes (i.e., turnover). This supports our expectation that *low-quality* employment relationships (i.e., PCB) will lead to negative processes and outcomes. Therefore, we expect the following:

**Hypothesis 1c**: *Work engagement will mediate the relationship between psychological contract breach and employee turnover intentions.*

### The Role of Unit Commitment Climate Level

Prior conceptual work on PCB (e.g., Morrison and Robinson, 1997) and some recent empirical work (e.g., Restubog et al., 2008, 2015) has argued that the social context could play a significant role in how individuals interpret PCB by either buffering or exacerbating PCB’s effects. For example, to make sense of a perceived PCB individuals can look to their social context, reasoning along the lines of “if all my colleagues are committed to the organization, the organization cannot be breaking promises on purpose too often” (cf. Kim et al., 2009). Therefore, we go beyond an idiosyncratic perspective in this study, and examine shared perceptions of a unit’s commitment climate, thereby answering recent calls for more “social PCB research” (e.g., Laulié and Tekleab, 2016).

To build a sound basis for the current study, as well for future research, we integrated recent developments in the unit climate literature (e.g., Cole et al., 2011) to delve deeper into the foundations of social context. Unit climate refers to shared perceptions within a unit (Schneider and Reichers, 1983), often referring to a type of “focused climate” approach specifically adapted to a certain type of climate (Lee and Dalal, 2016), such as psychological safety climate (Koopmann et al., 2016). In terms of PCB, we argue that especially *commitment climate* is important for understanding how employees make sense of their environment when experiencing PCB. Organizational commitment – which can be characterized as a dynamic summary of evaluative affect, cognitions about the organization, and a pledge to serve and enhance the organization’s purposes (Solinger et al., 2015) – has been central in prior PCB research (e.g., Zhao et al., 2007; Tomprou et al., 2015), and thus allows us to integrate our research on unit climate with the existing PCB literature. However, the PCB research thus far has studied commitment as an individual-level construct, whereas scholars from other fields have recently started to emphasize that work-related attitudes such as commitment are likely to become shared by members of a unit (Nishii et al., 2008). More specifically, Nishii et al. (2008) argue that unit-level commitment is likely to occur because unit members gain mutual experiences that are different from non-unit members, because they have reciprocal interactions that form collective perceptions of the organization, and because units establish distinct attraction–selection–attrition dynamics. Based on the focused unit climate literature and the unit-level commitment literature, we thus argue that unit commitment climate is a key factor in PCB processes. Moreover, commitment climate is especially important in relation to the dependent variable (i.e., turnover intentions) and the mediator (i.e., engagement). In deciding to put in one’s best effort, or contemplating leaving their organization or not, a relevant question for employees to ask themselves is how important their organization is to them, and how committed they feel to be to their organization. Thus, commitment climate is suited for our investigation of PCB, engagement, and turnover intentions as it denotes the relationships of an employee with the organization as well as coworkers’ relationship with the organization.

Especially *affective* commitment (Allen and Meyer, 1990; Meyer and Allen, 1991) is relevant with regard to PCB, because it is an emotional form of commitment: experiencing a PCB is primarily about expectations and emotions rather than cognitions (i.e., normative or continuance commitment). Hence, when examining effects of PCB it makes more sense to study affective response to this PCB compared to cognitive and normative forms of commitment (cf. Hansen and Griep, 2016). Additionally, also in terms of unit climate it is essential to examine the general sense of affective commitment among coworkers in one’s unit, as prevalent manifestations of commitment in a unit may serve as an influential guide and strong signaling function for employees for determining their own engagement with their job. This is in line with the team commitment literature (e.g., Drach-Zahavy and Freund, 2007), which states that teams can have a shared orientation in their commitment toward the organization, and that this team-level commitment can subsequently interact with individual team member’s perceptions and attitudes. We argue that this is especially so for PCB, as PCB triggers employees to question their own loyalty toward their organization (Rousseau, 1995) and to make sense of this PCB, employees can look around in their unit to see how loyal and committed their coworkers are toward their organization. Hence, we argue that investigating the unit’s commitment climate *level* – which is the mean shared perceptions among unit-members of the psychological bonds they have with their organization – is important.

More specifically, the effects of unit climate level on PCB can be understood by looking at social information processing (SIP) theory (Salancik and Pfeffer, 1978; Ho et al., 2006), which states that individuals adapt their attitudes, behaviors, and beliefs based on their social context. Experiencing PCB is a highly ambiguous event because it is a complex assessment of implicit agreements made between employee and employer (Morrison and Robinson, 1997). Therefore, individuals might find it difficult to fully comprehend this by themselves. The social environment in the unit provides individuals with cues on how to interpret – and make sense of – events, and about what would be appropriate attitudes and opinions. Drawing from SIP theory, we argue that unit climate commitment level significantly impacts individual attitudes and behavior because units create a shared perception of reality through their group processes (Salancik and Pfeffer, 1978). In terms of PCB, once individuals start interpreting their perceived breach, they will use their social context to assess how important their breach is (Morrison and Robinson, 1997), thereby impacting the subsequent effect on their work engagement. Following SIP theory, if the average commitment climate level is high in a particular unit, it illustrates a positive attitude toward the organization among one’s coworkers. Commitment is important as it is a crucial factor for employee motivation and well-being (e.g., Meyer and Maltin, 2010). Thus, when employees experience PCB, and they see that their coworkers in the unit are generally highly committed to the organization, this might act as a signal that the organization is not necessarily to blame for the perceived breach, or that it is less significant than initially felt. Following from this, the PCB may be attributed to factors outside the control of the organization or as a one-off event (Kim et al., 2009). Hence, in line with COR theory, this would imply that the perceived resource loss will be smaller and that the possible loss of future resources would be assessed to be less likely (Hobfoll, 2002). Thus, we expect that when commitment climate level is high, the detrimental effects of contract breach on work engagement will be buffered.

**Hypothesis 2**: *Commitment climate level will moderate the negative relationship between PCB and work engagement such that this relationship is weaker when commitment climate level is high as opposed to low.*

### The Role of Unit Commitment Climate Strength

Although, as discussed above, the climate level is important, it has recently been argued that solely focusing on climate level provides only half of the picture, as researchers have reasoned that it is also important to investigate the climate’s *strength* (e.g., Cole et al., 2011). This is important to account for the possibility that members within a unit differ in their agreement about their perceptions of the climate (Dawson et al., 2008). Within the climate literature, an increasing number of studies has indicated that climate strength is a valuable way of assessing unit climate effects on outcomes, showing, for example, that high climate strength is associated with increased customer satisfaction (Schneider et al., 2002) and team performance (González-Romá et al., 2009). However, akin to climate level, climate strength has not yet been systematically connected to PCB, and we therefore decided to do so by explicitly integrating the PCB and climate literatures. In the text leading to Hypothesis 2 we drew on COR and SIP theory to discuss the importance of climate level for PCB and below we will use the same theories to argue why climate strength might also be important for PCB.

Social information processing theory (Salancik and Pfeffer, 1978) states that SIP will have a more powerful effect on individual unit members when the level of unanimity is high. That is, the more unanimous unit members are in their beliefs, the more strongly unit commitment climate will interact with PCB on the unit members’ work engagement. More specifically, if climate strength is low, which can be referred to as a “weak situation” as there is not much agreement within the unit (Meyer et al., 2010), the unit context offers mixed, confusing, and/or conflicting signals that will not help individuals to make sense of the information provided to them. In such cases, the unit context fails to provide the employee with clear and unanimous information on how to interpret the PCB and thereby the social context cannot buffer its consequences. Contrarily, when unanimity is high, there is a “strong situation” (Meyer et al., 2010) in which there is a clear shared perception within the unit. This clarity and uniformity will facilitate the individual to make sense of the PCB, and the social cohesion within the unit can thus act as a buffer in the resource depletion process as described by COR theory. In sum, following SIP theory and the argument that strong situations will help to guide individual behavior, we expect that high commitment climate strength will buffer the negative relationship between PCB and work engagement.

**Hypothesis 3:** *Commitment climate strength will moderate the negative relationship between PCB and work engagement such that this relationship is weaker when commitment climate strength is high as opposed to low.*

### The Interaction Between Commitment Climate Level and Strength

Research on unit climate is valuable for building on recent calls on “social PCB research” (Laulié and Tekleab, 2016), yet in the emerging social PCB research most studies have taken an “either-or-approach” (instead of an “and-and-approach”). Within the climate literature, only a few recent climate studies have examined the interaction between both constructs, arguing that climate strength might enhance the effects of climate level on outcomes. These climate studies have claimed that a high climate level is most effective when climate strength is also high. For example, González-Romá et al. (2009) showed that team performance could only be enhanced in a situation where climate had a high level *and* a high strength. Similarly, De Jong and Bruch (2013) showed that leadership climate level *and* strength affected organizational performance. Hence, recent advances in the climate literature indicate that research should investigate both climate level and strength simultaneously as they might interact.

Social information processing theory (Salancik and Pfeffer, 1978) supports this notion, as individuals will use their social environment to form their attitudes and beliefs. Relating this to our discussion on PCB, we have argued that higher overall level of commitment in the unit (H2) and higher level of unanimity in the unit (H3) could both buffer the negative effect of PCB on work engagement. We maintain that these two unit climate concepts could have unique effects, yet it seems likely that the strongest buffering effect would occur in a situation in which *both* the average level of commitment climate and the unanimity regarding the unit climate are high (Schneider et al., 2002). That is, in a situation where unit members are all highly committed to the organization, this shared reality of high affective commitment to the organization will help the individual to interpret the PCB as less significant and stressful, thereby buffering the resource depletion process (cf. COR theory) that occurs after PCB. More specifically, because, as explained above, PCB is highly ambiguous, strong situations characterized by high affective comments level *and* strength will provide individuals with clear social cues about whether the breach they experienced might have been a one-off event, and/or how likely it is to occur again. In cases when unit members are homogenously highly committed to the organization, individuals are likely to conclude that their PCB was a relatively minor one-off accident, thereby reducing the resource depletion process. In sum, our final hypothesis is a three-way interaction in which we expect that especially in a situation with high commitment climate level *and* high commitment climate strength, the negative relationship between PCB and work engagement will be buffered.

**Hypothesis 4**: *The negative relationship between PCB and work engagement will be buffered when both commitment climate level and commitment climate strength are high.*

## Materials and Methods

### Procedure and Participants

The study was conducted in three Dutch health care organizations, which in total consisted of 36 different units, all of which provided care for the elderly. These units represented different geographical locations across the organizations, which until recently before the data collection, operated as small self-managed nursing homes. Due to mergers, many of these nursing homes became part (i.e., a unit) of larger health care organizations. Paper-and-pencil questionnaires were sent to all employees across the units, and managers were instructed to encourage participation in the research among the employees. Postboxes were placed in all locations, where employees could return their questionnaire. Anonymity and confidentiality were guaranteed by the researchers. After merging all data together, the dataset consisted of 1,272 employees in 36 different units (average response rate 54%), which is enough to perform multilevel modeling according to Mcneish and Stapleton (2016). On average, the mean number of employees in a unit was 35 (*SD* = 9.09).

With regard to research ethics, we did not seek approval from an ethical committee as the survey research that we performed was exempt from such approval in the country in which the study was performed (i.e., Netherlands) and by the institutions leading this project. All research participants were informed in an introductory explanation of the survey that they would formally agree to participate in the research by filling out the survey, thereby giving informed consent if they chose to participate. All participants were informed that their participation was completely voluntary and that they could quit at any time.

In total, 91% of the employees were female, which was representative for the total population, and is not uncommon in the healthcare sector. The average age of the respondents was 43.52 years, on average they had 1.10 children living at home, and 80% had a permanent contract. Participants worked on average 24 h a week, mean organizational tenure (at the current organization and one of the predecessors) was 10.50 years, and mean functional tenure 7.94 years. 35.6% of the participants had finished primary school or some vocational training, 47.4% had finished intermediate vocational training, and 17% had finished higher vocational training or a university degree.

### Measurement Instruments

*Psychological contract breach* (α = 0.85) was measured using the five-item global breach measure of Robinson and Morrison (2000). This individual-level scale assesses the extent to which the organization fulfilled or broke its obligations to the respondent. An example item was: “[The organization] has broken many of its promises to me even though I’ve upheld my side of the deal” (*1* = not at all; *5* = to a very great extent).

*Work Engagement* (α = 0.92) was measured using six items from the UWES measure (Schaufeli and Bakker, 2004), indicating vigor and dedication to the job. Vigor and dedication are considered the core dimensions of work engagement (González-Romá et al., 2006). An example item is: “I am enthusiastic about my job” (*1* = never; *7* = always).

*Turnover intentions* (α = 0.88) were measured with five items from Aquino et al. (1997), an example being: “I am actively searching for a job outside [organization]” (*1* = not at all; *5* = to a very great extent). The measure indicates employees’ intentions to leave their organizations and their activity in searching a new job.

*Commitment climate* (α = 0.83) was measured using individualized responses to the eight-item affective commitment measure of Allen and Meyer (1990), an example being: “This organization has a great deal of personal meaning for me” (*1* = not at all; *5* = to a very great extent). To obtain the *commitment climate level* score, we aggregated the individual responses to the unit level via a direct consensus approach in which individual response were aggregated to the unit level (Chan, 1998), because this is the most appropriate when studying job-related attitudes such as commitment and engagement (Wallace et al., 2016). As commitment climate is assumed to be a construct that is shared among members within a unit, we calculated ANOVA, ICC1, and ICC2 statistics to assess whether it was appropriate to do so (Van Mierlo et al., 2009). Between unit-variance was significant [*F*(1236, 35) = 3.45, *p* < 0.001]. ICC1 was 0.02, and ICC2 was 0.71. We also calculated the *r*_wg_ score to justify aggregation, which was 0.91 (range 0.87–0.97) and thus clearly meets criteria for multilevel aggregation (Klein and Kozlowski, 2000). Hence, although the ICC1 was on the low side (cf. Lebreton and Senter, 2008), our sample is large and ICC2 and *r*_wg_ statistics were good (Bliese, 2000). Moreover, various other studies have reported similar statistics (e.g., Carter et al., 2015; Ou et al., 2018), and there is thus support for our approach of calculating commitment climate level by taking the unit mean.

*Commitment climate strength* was measured using the variability of the unit members’ responses to the commitment measure. The scores were calculated on the basis of the variability of participants’ perceptions of commitment within their unit. This was done by calculating the standard deviation of the commitment scale within each unit, as suggested by Roberson et al. (2007). Subsequently, to increase interpretation, and following the advice of Cole and Bedeian (2007), we converted these heterogeneity scores (i.e., higher scores indicate higher heterogeneity) to consensus scores by multiplying them by −1 (i.e., higher scores indicate higher consensus). Hence, commitment climate strength is a unit-level construct (i.e., level 2) in which higher scores represent higher consensus within the unit.

### Control Variables

In our analyses, we took into account the three parent organizations by using two dummy variables at the unit-level. We used these dummies, as the correlations (Table 1) showed significant correlations between organization and climate level and strength. This was not surprising to the authors, as in preparing and conducting the research in the three organizations, the authors observed three different organizational cultures and management styles, which likely affected the ways employees felt and built commitment to their organizations and we compensate for this via these dummies. In addition, at the individual-level we included gender (*0* = male, *1* = female), employee age (in years), educational level (measured using the highest finished degree; *0* = primary education; *6* = university degree), total number of contractual weekly working hours, organizational tenure (in years), functional tenure (in years), and contract status (*0* = temporary contract; *1* = permanent contract) as these may be correlated with the outcomes (Griffeth et al., 2000; Crawford et al., 2010). The latter three were not significantly related to any of the outcome variables, and did not affect the significance of the main predictors. Hence, we decided to run the analyses without these control variables to preserve a good data to variable ratio (Becker, 2005). Moreover, as age tends to be correlated with organizational and functional tenure, it was appropriate to retain employee age in the analyses.

**Table 1:** Means, standard deviations, reliabilities, and correlations of the study variables.

Variable		Level	*M*	*SD*	AVE	1	2	3	4	5	6	7	8	9	10	11
1.	Org. Dummy 1	2	0.43	–	–	–										
2.	Org. Dummy 2	2	0.42	–	–	−0.74^∗∗^	–									
3.	Gender	1	0.91	–	–	0.01	−0.04	–								
4.	Age	1	43.52	10.69	–	−0.11^∗∗^	0.04	−0.08^∗∗^	–							
5.	Education	1	2.44	1.42	–	0.01	0.00	−0.27^∗∗^	−0.16^∗∗^	**–**						
6.	Working hours	1	24.17	9.84	–	0.05	0.01	−0.35^∗∗^	0.04	0.27^∗∗^	**–**					
7.	PCB	1	2.24	0.78	0.63	0.11^∗∗^	−0.09^∗∗^	0.05	0.01	−0.07^∗^	−0.01	(0.85)				
8.	Climate level	2	3.26	0.19	0.75	−0.67^∗∗^	0.34^∗∗^	−0.10^∗∗^	0.10^∗∗^	0.12^∗∗^	0.11^∗∗^	−0.14^∗∗^	(0.83)			
9.	Climate strength	2	−0.60	0.08	–	−0.57^∗∗^	0.40^∗∗^	0.00	0.08^∗∗^	−0.02	−0.04	−0.04	0.48^∗∗^	–		
10.	Work engagement	1	5.67	1.02	0.71	−0.05	0.04	0.02	0.11^∗∗^	−0.06^∗^	0.14^∗∗^	−0.26^∗∗^	0.10^∗∗^	0.01	(0.92)	
11.	Turnover intention	1	1.91	0.91	0.74	0.12^∗∗^	−**0**.06^∗^	0.00	−0.13^∗∗^	0.13^∗∗^	−0.02	0.37^∗∗^	−0.14^∗∗^	−0.08^∗∗^	−0.42^∗∗^	(0.88)

*Reliabilities are reported along the diagonal between brackets. N_level 1_ = 1,272; N_level 2_ = 36. Gender: 0 = male; 1 = female. Education 1 = primary education; 6 = university degree. ^∗^p < 0.05; ^∗∗^p < 0.01. PCB = psychological contract breach.*

### Strategy of Analysis

The study provided data at both the individual level (e.g., PCB), as well as the unit-level (e.g., commitment climate strength). Since the individual-level data are nested within units, multi-level analyses are required. Multilevel path analyses using MPlus 7 (Muthén and Muthén, 2012) were conducted to test the hypotheses. Independent variables were grand-mean centered and we used random intercept modeling (Hox, 2002). The multilevel path analyses allowed us to test the full model in one analysis, including the mediating effects, as well as the cross-level interactions effects of commitment climate level and commitment climate strength. Standardized coefficients will be reported as γ-values, and unstandardized coefficients will be reported as *b*-values.

## Results

### Correlations

Table 1 shows the means, standard deviations, and correlations among the study variables. Commitment climate level was positively related to commitment climate strength (*r* = 0.48, *p* < 0.01), indicating that in units with higher average levels of commitment, there was also more consensus among employees in their levels of commitment to the organization. However, there was no indication of multicollinearity of these measures, as they only have 23% common variance and thus have enough unique variance. Moreover, PCB was negatively related to work engagement (*r* = −0.26, *p* < 0.01), and positively to turnover intention (*r* = 0.37, *p* < 0.01), indicating the negative consequences contract breach might have for employees’ engagement and turnover intentions.

### Confirmatory Factor Analysis, Measurement Model, and CMV Tests

To assess the factor structure, we conducted a multilevel confirmatory factor analysis (M-CFA) of the multi-item scales under study. We used MPlus to conduct the analyses. Table 1 shows the results of the scale analyses. The proposed model with four-factors, including measures of PCB, work engagement, turnover intention, and affective commitment (as measured at Level 2) obtained a good fit (χ^2^ = 931.504, *df* = 121, *p* < 0.001; RMSEA = 0.073, SRMR within = 0.058, SRMR between = 0.081, CFI = 0.922, TLI = 0.905). All standardized loadings of the items were significant in relation to their indicators and above 0.40. Moreover, we also tested two alternative models, including a model with work engagement and turnover intention loading on one factor (to control for the alternative that there was only one overall job attitude). As Table 1 shows, all of these alternative models fitted significantly worse than our proposed model, indicated by differences greater than 0.002 in CFI (Meade et al., 2008). Hence, we conclude that the measurement model is valid and that, as expected, our measures are empirically different constructs.

Furthermore, we used the marker variable approach to test whether common method variance (CMV) affected the correlations of the study variables (Lindell and Whitney, 2001). We used number of children living at home (Range 0–10, *M* = 1.17) as a marker variable, as this construct was irrelevant to the hypotheses. We found small correlations between children and the main variables under study (ranging between −0.06 and 0.05). Number of children was only negatively related to working hours (*r* = −0.14, *p* < 0.01). Hence, there was no concern with empirical overlap between children and the main variables. We calculated correlations among the study variables while controlling for number of children (Lindell and Whitney, 2001), which produced very similar correlations as reported in Table 2, with no differences in significance of correlations. Hence, these analyses further showed that CMV was not affecting the results of our study.

**Table 2:** Multilevel confirmatory factor analysis.

Model	χ^2^	*df*	RMSEA	SRMR within	SRMR between	CFI	TLI
Four-factor	931.504	121	0.073	0.058	0.081	0.922	0.905
Three-factor	3328.309	123	0.143	0.139	0.081	0.691	0.629
Two-factor	5610.496	124	0.187	1.82	0.081	0.472	0.370

*Four-factor: PCB, work engagement, turnover intention (all level 1), and commitment climate (level 2). Three-factor: PCB, job attitudes (work engagement and turnover intention), and commitment climate. Two-factor: work attitudes (PCB, work engagement, and turnover intention), and commitment climate.*

Finally, we also calculated average variance extracted to test for the proportion of variance that is explained due to random error (Fornell and Larcker, 1981), which is another test for the validity of the measures. The average variance extracted scores should be >0.50, and Table 1 presents our multi-item measures, for which all average variance extracted scores were 0.63 or higher, thereby supporting the convergent validity and reliability of the measures. In sum, these three analyses demonstrate that the variables represent significantly different constructs and that CMV is unlikely to affect the results.

### Tests of Hypotheses

Table 3 shows the results of the multilevel path analyses and the multi-level fit statistics were acceptable (χ^2^ = 138.94, *df* = 7, *p* < 0.000; SRMR within = 0.039, SRMR between = 0.104). In line with Hypothesis 1a, contract breach was negatively related to work engagement (standardized coefficient γ = −0.27, *p* < 0.001), and in line with Hypothesis 1b, work engagement was negatively related to turnover intentions (γ = −0.41, *p* < 0.001). Finally, in line with Hypothesis 1c, the indirect effect from PCB to turnover intentions via work engagement was also significant (γ = 0.13, *p* < 0.001), thereby fully supporting Hypothesis 1a, 1b, and 1c. In sum, these results show that work engagement mediates the relationship between PCB and turnover intention.

**Table 3:** Multilevel path analysis of PCB on work engagement and turnover intention, and the moderating roles of commitment climate level and strength.

Variables	Level	Work engagement	Turnover intentions

		γ (*SE*)	γ (*SE*)
**Control variables**			
Organization dummy 1	2	−0.36 (0.38)	0.95 (0.51)
Organization dummy 2	2	−0.13 (0.15)	0.39 (0.30)
Gender	1	0.07 (0.11)	0.01 (0.13)
Age	1	0.08 (0.03)^∗∗^	−0.08 (0.02)^∗∗∗^
Education	1	−0.11 (0.05)^∗^	0.08 (0.06)
Working hours	1	0.18 (0.04)^∗∗∗^	0.00 (0.04)
**Independent variables**			
Psychological contract breach (PCB)	1	−0.27 (0.04)^∗∗∗^	−
Commitment climate level	2	0.11 (0.62)	−
Commitment climate strength	2	−0.44 (0.27)	−
**Interactions**			
PCB ^∗^ Climate level	1^∗^2	0.03 (0.04)	−
PCB ^∗^ Climate strength	1^∗^2	0.06 (0.02)^∗^	−
Climate level ^∗^ Climate strength	2^∗^2	0.02 (0.09)	−
PCB ^∗^ Climate level ^∗^ Climate strength	1^∗^2^∗^2	0.04 (0.02)^∗^	−
**Mediator**			
Work engagement	1	−	−0.406 (0.05)^∗∗∗^
χ^2^		−	139.94^∗^
*df*		−	135
*R*^2^ (within level)		0.11	0.19
*R*^2^ (between level)		0.27	0.69

*^∗^p < 0.05, ^∗∗^p < 0.01, ^∗∗∗^p < 0.00.*

**FIGURE 2:**
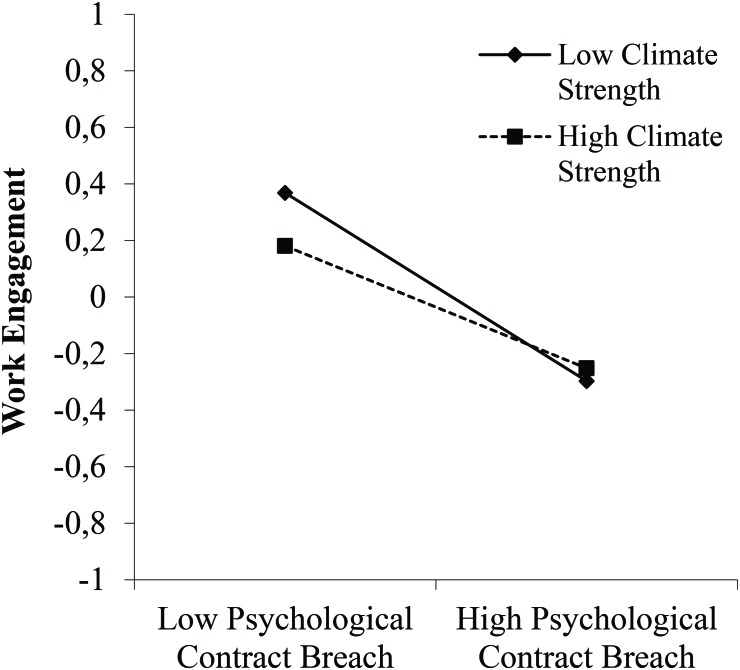
The interaction between PCB and commitment climate strength in relation to work engagement.

Hypothesis 2 predicted that commitment climate level would moderate the relationship between PCB and work engagement. This hypothesis was not supported, as the interaction was non-significant (γ = 0.03, *ns*). Hypothesis 3 predicted an interaction effect of commitment climate strength in the relation between PCB and work engagement. This cross-level interaction was significant (γ = 0.06, *p* < 0.05). Figure 2 shows the interaction pattern. The slope was strongly negative for low consensus units (*b* = −0.43, *p* < 0.001), while the slope was less negative for high consensus units (*b* = −0.28, *p* < 0.001). These slopes are supportive of Hypothesis 3, and show that the negative relationship between PCB and work engagement was buffered in units with more homogenous commitment climates. Taking a closer look at Figure 2 shows that it was especially in the context of low PCB that the two slopes diverged and that engagement was actually higher among employees in low climate strength units. Overall, this suggests that climate strength especially mattered in the context of increasing PCB, because then high climate strength buffers the relationship of PCB with engagement.

**FIGURE 3:**
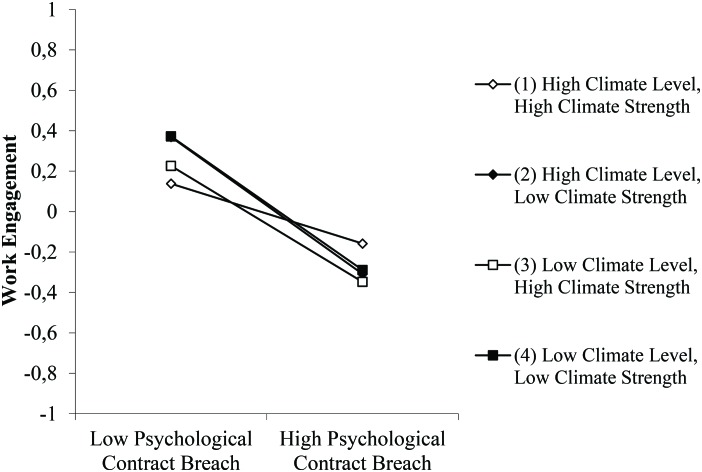
The three-way interaction between PCB, commitment climate level, and commitment climate strength in relation to work engagement.

Hypothesis 4 predicted a three-way interaction between PCB, commitment climate level, and commitment climate strength in relation to work engagement. This interaction effect was significant (γ = 0.04, *p* < 0.05), supporting Hypothesis 4. To explore the three-way interaction in more detail we drew the slopes (Figure 3) and also statistically analyzed each of them. The slope representing units with high climate level and high climate strength was non-significant (*b* = −0.19, *ns*), indicating that PCB did not significantly damage work engagement under this condition. For units with low climate level and high strength the slope was negative and approached significance (*b* = −0.37, *p* < 0.10). The slopes for units with high climate level and low strength (*b* = −0.43, *p* < 0.05), and for units with low climate level and low strength (*b* = −0.42, *p* < 0.05) were also negative and significant. This provides evidence that especially within units with both high commitment level *and* high commitment strength, the relationship between PCB and work engagement was buffered. Further slope difference tests revealed that the “high level/high strength” slope differed significantly from the “high level/low strength” slope (*t* = 2.99, *p* < 0.01), the “low level/high strength” slope (*t* = 2.30, *p* < 0.05), and the “low level/low strength” slope (*t* = 1.99, *p* < 0.05). The other three slopes did not differ significantly from each other. The three-way interaction was also significantly related to turnover intention via engagement (indirect effect *b* = −1.16, *p* < 0.05), providing evidence for a moderated-mediation effect in which the three-way interaction was indirectly related to higher turnover intention via work engagement. In sum, as expected in Hypothesis 4, the main driver behind the three-way interaction effect is the high commitment climate level *and* high commitment climate strength situation, as then PCB does not significantly affect work engagement anymore.

Finally, to assess the robustness of our main model, we performed alternative analyses with regard to potential interaction between only PCB, commitment climate level, commitment climate strength, and turnover intentions. Our results showed no significant two-way interaction effect of PCB with climate level (γ = 0.00, *ns*) or climate strength (γ = 0.03, *ns*) with turnover intention. The three-way interaction between PCB, climate level, and climate strength was also unrelated to turnover intentions (γ = 0.04, *ns*). This further supports the notion that work engagement mediates the relationship between PCB and turnover intentions, and that the effect of commitment climate level and strength manifests via work engagement rather than directly relating to turnover intentions. Our explanation for the non-occurrence of the interaction effect in relation to turnover intention resides in our theoretical model. Enjoyment of one’s job (engagement) is a more proximal outcome of a PCB than the more distal outcome turnover intention. In other words, PCB will first have emotional effects on job attitudes (i.e., engagement), and only then affect other outcomes such as intentions to leave the organization (Morrison and Robinson, 1997; Zhao et al., 2007). It is therefore also more likely that in responding to PCB, climate will be more likely to impact proximal outcomes than more distal outcomes, as with more distal outcomes other factors may play a role, such as job opportunities beyond one’s organization. Hence, it is more likely that proximal outcomes will be affected than more distal outcomes.

## Discussion

The impetus for this study were recent calls for more research on the role of social context in PCB (e.g., Laulié and Tekleab, 2016), to which we heeded by integrating contemporary knowledge from the unit climate literature (e.g., González-Romá et al., 2009) into the PCB scholarly discussion. Drawing from COR theory (Hobfoll, 2001) and SIP theory (Salancik and Pfeffer, 1978), we examined whether unit commitment climate *level* (i.e., a high mean score of organizational commitment among unit members) and *strength* (i.e., a high consensus among unit members about their organizational commitment) could each buffer the detrimental effects of PCB on work engagement and, subsequently, turnover intentions. To assess these buffering effects, we first tested our core mediation model, which was supported as we found that PCB related to reduced work engagement and subsequently also to increased turnover intentions. Second, the results indicated that unit commitment climate played an important part in employees’ interpretation of PCB. Specifically, whereas commitment climate level did not buffer the negative effect of PCB on work engagement, commitment climate strength did. Third, we found a three-way interaction demonstrating that the detrimental effects of PCB on work engagement is especially buffered when *both* commitment climate level and strength are high. Finally, this three-way interaction was also indirectly related to turnover intentions via work engagement. This is, to the best of our knowledge, the first study that explicitly integrates the literatures on PCB and unit climate, showing that the social context of employees is influential and more complex than often assumed in PCB research, as we found that climate level *and* strength jointly play a crucial role in how employees deal with PCB.

### Implications for Research and Theory

Our findings have several theoretical implications. First, whereas PCB scholars have highlighted the importance of the social context in the aftermath of contract breach (e.g., Morrison and Robinson, 1997), and some studies have included contextual variables when examining PCB (e.g., Epitropaki, 2013), no studies to date have theorized about – or examined – the role of climate *strength* (i.e., the variance in scores on a contextual factor) in empirical studies on PCB. What makes our findings unique, is that we integrated knowledge on unit climate level and unit climate strength (e.g., Cole et al., 2011), and incorporated this knowledge into one conceptual model based on COR theory and SIP theory, to demonstrate that the negative impact of PCB on worker outcomes is especially buffered when *both* commitment climate level *and* strength are high. In sum, these findings build on recent conceptual work on the role of social context in psychological contracts (Laulié and Tekleab, 2016) by illustrating the importance of incorporating social contextual elements – such as unit climate – in research on PCB and we add to that the insight that it is important to be very clear and explicit about both climate level and strength.

Second, in applying the principles of COR theory (Hobfoll, 2001) and SIP theory (Salancik and Pfeffer, 1978) to PCB, we introduced a novel way of thinking about the processes that unfold after PCB occurs. We borrowed the principle of resource loss cycles from COR theory as being a potentially threatening effect of a PCB for employee well-being and performance, thereby arguing that a PCB can be considered a resource loss as promises and support by the organization (i.e., a resource) is perceived to be lost. While some other authors have considered COR theory in psychological contract research (e.g., Bal et al., 2013; Garcia et al., 2018), its application is still quite new in this literature. Furthermore, in attempting to empirically examine the role of social context in psychological contract research, we integrate SIP theory and borrow its principles about the role of social cues in individual sensemaking and attitude forming, as well as the role of “strong situations” (i.e., high unit commitment climate level and strength) (Meyer et al., 2010) in making sense of contract breach. Besides these separate contributions to COR and SIP theory, our study suggests an interesting possibility for combining both theories, namely that unit commitment climate level and strength have buffering effects on the consequences of PCB because they provide resources themselves. In this sense, SIP in terms of having unit members that are all highly committed to the organization may provide employees with the necessary (social) resources to compensate for another resource loss (i.e., the organizationally induced resources loss due to PCB). Thus, our study indicates that COR theory (Hobfoll, 2001) and SIP theory (Salancik and Pfeffer, 1978) may be closely related to each other when individuals experience a PCB and future research could use this to investigate if this also applies in other circumstances.

Finally, this study contributes to research on unit climate by emphasizing the “and–and” approach rather than the “either-or” approach. More specifically, most studies examining unit climate have either looked at climate level (e.g., Spell and Arnold, 2007) or at climate strength (González-Romá et al., 2009). Recently, Cole et al. (2011) argued and showed that especially their interaction offers important new insights into the role of unit climate. In line with Cole et al. (2011), we also demonstrate that climate level and climate strength interact, and we expand the empirical basis by investigating new relationships, namely the buffering the negative effects of PCB on work engagement. In conclusion, our study adds support to the idea that theorizing on social context should incorporate both aspects of unit climate rather than only one, and by using both COR and SIP theory we have provided new theoretical angles for doing so not only for the PCB literature, but also the unit climate literature.

### Limitations and Suggestions for Future Research

The current study has several strengths, such as using multilevel path analyses in a large sample of employees, and exploring new aspects of the role of social context in PCB by integrating the unit climate literature into PCB research. Nevertheless, there are also some limitations. The first important limitation concerns the cross-sectional nature of our data, which makes it impossible to infer cause-and-effect relationships among our study variables. We do ground our hypotheses firmly in the existing literature, yet we cannot fully prove that work engagement arises from PCB and then causes turnover intentions due to our dataset. Therefore, future research could use our theorizing and employ longitudinal data to assess the causal relationships in more depth.

Second, our data consisted of self-reports and the possibility of biases therefore has to be investigated. First, scholars have argued that common methods biases are unlikely when significant interaction effects are found (Evans, 1985). Additionally, since we had a multilevel research design, we aggregated individual scores on commitment level and strength to create composite unit-level scores, and the use of multiple methods and multiple levels further reduces concerns about biases (e.g., Podsakoff et al., 2003). Finally, our research is in line with the notion that constructs such as PCB, work engagement, and turnover intentions are most reliably measured by self-perceptions (Mäkikangas et al., 2004) and our CFA and CMV tests supported this decision, by showing the convergent and discriminant validity of our constructs. Yet, future research might use multi-rater data and/or include objective data to further develop our new framework.

In this study, we specifically chose unit commitment climate as our focal measure, because the commitment that employees and their coworkers have toward their organization is likely to impact their responses to PCB (Zhao et al., 2007). Although this is an important first step in examining the role of unit climate after PCB, there are other types of focused climates (Lee and Dalal, 2016) that would be interesting to study in tandem with PCB. For example, voice climate (Frazier and Bowler, 2015) and justice climate (Spell and Arnold, 2007) could be crucial in determining an employee’s response to PCB. More specifically, if PCB occurs in units that have a climate that is characterized by fairness in procedures and outcomes (i.e., justice climate) and/or by feeling free to speak up in case of problems (i.e., voice climate), this could buffer the negative effects of PCB. In sum, future studies can build on this study by examining different types of unit climates affecting PCB responses and we hope our integration of the PCB and unit climate literatures provides a useful starting point.

### Implications for Practice

An important practical implication of this study is that consistency in how committed employees are to their organization, especially when combined with an overall high level of commitment, is an important way of retaining valuable employees and enhancing their well-being at work. Accordingly, HRM practices and policies could focus on nourishing the commitment of their staff to prevent potential PCB occurrences of having a detrimental effect on their work engagement and their intentions to leave the organization. For example, Toh et al. (2008) describe commitment-maximizing HR bundles, which means that organizations invest in the full range of high performance HR practices in order to ensure maximum motivation and commitment among their staff. This includes a careful selection and socialization process, as well as providing employees with opportunities to contribute effectively (Toh et al., 2008). Other important elements via which commitment can be maximized, include providing internal development opportunities and enhancing procedural justice in the organization (Boxall and Macky, 2009; Boon and Kalshoven, 2014), as well as focusing on employee empowerment, for example by providing job rotation and developmental feedback (Whitener, 2001). If organizations would employ such HR bundles and make sure that they include all of their employees rather than only the high potentials (i.e., use an *inclusive* approach to HRM instead of an *exclusive* one; Dries, 2013), this could be an important way to minimize the potential detrimental effects of PCB.

Our study also has implications for the leadership in organizations. As our findings indicate that the negative effects of PCB can be diminished in a social context characterized by unanimity among unit members, it follows that especially group-focused leadership behaviors (Wang and Howell, 2010) are appropriate. Research has shown that differentiated leadership behaviors – that is, treating some individuals in a group differently than others – can cause negative outcomes (Wu et al., 2010). As a consequence, if leaders want to prevent negative effects of PCB, they could best employ group-focused leadership behaviors that enhance unanimous commitment among their followers.

Finally, the underlying theory of our study indicates *why* the above mentioned practical implications would be effective, namely because they are both likely to induce high levels of commitment among *all* workers. As a consequence, those workers would obtain clear and unambiguous information from the organizational as well as from their social context which makes it clear that their resources are protected, thus diminishing the potential harmful effects of PCB. As our findings show, this would nourish work engagement and would reduce turnover intentions, thereby allowing organizations to create an engaged workforce that is focused on staying in the organization (Turnley et al., 2003; Cappelli and Keller, 2013). Consequently, since research has shown that PCB occurs among a high proportion of employees and its negative effects are difficult to repair (Solinger et al., 2016), we would advise practitioners to focus on creating high commitment among *all* employees.

## Data Availability

The datasets generated for this study can be obtained from the second author upon request.

## Author Contributions

JA: theorizing, analyzing, writing, and coordinating. MB: theorizing, analyzing, and writing. SD: theorizing and writing.

## Conflict of Interest Statement

The authors declare that the research was conducted in the absence of any commercial or financial relationships that could be construed as a potential conflict of interest.
